# Genetic and Phenotype Analysis of a Chinese Cohort of Infants and Children With Epilepsy

**DOI:** 10.3389/fgene.2022.869210

**Published:** 2022-04-27

**Authors:** Zhang Chuan, Cai Ruikun, Li Qian, Mei Shiyue, Hao Shengju, Yuan Yong, Li Haibo, Xiao Neng, Zhao Yong, Xue Huiqin, Wang Weijia, Hui Ling, Zhou Bingbo, Qinghua Zhang, Wang Yan, Cao Zongfu, Ma Xu

**Affiliations:** ^1^ National Research Institute for Family Planning, National Human Genetic Resources Center, Beijing, China; ^2^ Gansu Province Medical Genetics Center, Gansu Provincial Clinical Research Center for Birth Defects and Rare Diseases, Gansu Provincial Maternity and Child-Care Hospital, Lanzhou, China; ^3^ Graduate School of Peking Union Medical College and Chinese Academy of Medical Sciences, Beijing, China; ^4^ Department of Intensive Care Unit, Henan Provincial Key Laboratory of Children’s Genetics and Metabolic Diseases, Children’s Hospital Affiliated to Zhengzhou University, Zhengzhou, China; ^5^ Zhongshan City People’s Hospital, Affiliated Zhongshan Hospital of Sun Yat-Sen University, Guangzhou, China; ^6^ The Central Laboratory of Birth Defects Prevention and Control, Ningbo Women and Children’s Hospital, Ningbo, China; ^7^ Department of Pediatric Neurology, Chenzhou First People’s Hospital, Chenzhou, China; ^8^ Foshan Women and Children Hospital, Foshan, China; ^9^ Children’s Hospital of Shanxi, Women Health Center of Shanxi, Taiyuan, China

**Keywords:** infant and children epilepsy, variant, phenotypes, WES, genetics counseling

## Abstract

**Background:** Epilepsy in childhood is a common and diverse neurological disorder. We conducted a genetic and phenotype analysis of a Chinese cohort of infants and children with epilepsy.

**Methods:** We conducted a pedigree analysis of 260 Chinese patients with epilepsy onset during infancy or childhood by whole exome sequencing (WES).

**Results:** Of the 260 probands analyzed, a genetic diagnosis was established in 135 patients. One-hundred eighty-eight phenotypes were detected in those 135 positive/likely positive patients, 106 patients had more than two phenotypes, and 67 patients had more than three phenotypes. A total of 142 variants of 81 genes were detected among the positive/likely positive patients. Among these 142 variants, of which 87 of 66 genes were novel.

**Conclusion:** Our findings extend the variant spectrum of genes related to epilepsy. Our results will be useful for genetic testing and counseling for patients with epilepsy.

## Introduction

Epilepsy is a group of common and diverse neurological disorders characterized by spontaneous, unprovoked, recurrent seizures (Deng et al., 2014). The etiology of epilepsy is complex, it is reported that many genetic factors are associated with epilepsy (Deng et al., 2014). There are two main types of genetic factors: (1) genes associated with primary epilepsy syndrome, in which epileptic seizures are the main clinical feature; and (2) genes associated with brain development disorders that produce epileptic seizures and other clinical features, such as epileptic encephalopathy, which is a heterogeneous category of destructive epileptic disorders characterized by frequent severe epilepsy indicated by interictal epileptiform discharges on an electroencephalogram (EEG), and progressive cognitive and neuropsychological deterioration (Poduri and Lowenstein, 2011; Deng et al., 2014). The pathogenic effects of these genetic variants involve synaptogenesis, pruning, neuronal migration and differentiation, neurotransmitter synthesis and release, alterations of the structure and function of membrane receptors and transporters (Zupanc, 2009; Galanopoulou and Moshé, 2009), and accumulation of harmful metabolites in the brain.

Epilepsy is one of the most common neurological diseases seen in children, with the highest incidence in the first year of life (Fine and Wirrell, 2020). The seizure semiotics in infants and children are simpler than those in adolescents and adults (Park et al., 2020). The causes and clinical spectrum of epilepsy are extremely wide-ranging in children (Guerrini, 2006). Understanding common childhood epilepsy syndromes is valuable when approaching the diagnosis and management of a child presenting with seizures (Carney et al., 2005). Syndrome-oriented clinical and EEG diagnosis and improved etiological diagnosis, especially that supported by neuroimaging, has helped to clarify the diversity of epilepsy in children and has improved epilepsy management (Carney et al., 2005). Moreover, different epilepsy syndromes sometimes associated with the same genetic variant or variants in different genes can result in a similar phenotype, and the complexities of the genotype-phenotype correlation increase the difficulty of accurate clinical diagnosis.

Here, we recruited 260 Chinese infants and children with epilepsy and their family members. Whole exome sequencing (WES) was performed on the probands in each family to examine the genetic etiology.

## Patients and Methods

### Patients

We recruited 260 patients with infancy or childhood-onset epilepsy at 7 hospitals in 7 provinces in China. Age of onset of all the patients are less than 3 years old, and the age of diagnosis ranged from 3 days to 26 years old. A team of genetic counselors and neurologists at each hospital reviewed each case’s clinical data and testing, including the patient’s seizure symptoms, EEG findings, and brain imaging reports. Epilepsy caused by birth trauma and traumatic brain injury was excluded, and the diagnostic criteria were based on the criteria of the International League Against Epilepsy (Fisher et al., 2017). The clinical characteristics of the probands are summarized in Table 1. This study was undertaken according to the tenets of the Declaration of Helsinki 1975 and its later amendments. The study protocol was approved by the Ethics Committee of the National Research Institute for Family Planning (Beijing, China). Written informed consent was obtained from all study participants or their legal guardians.

**TABLE 1:** General clinical features of the probands in this study.

Characteristics	Number/range
Male	155
Female	105
Age of onset	3 Day-3 years old
Age of diagnosis	3 Day-26 years old
Isolated epilepsy	67
Epilepsy with development delay	34
Epilepsy with intellectual disability	24

### Genomic DNA Preparation

Genomic DNA was extracted from peripheral blood samples (2–3 ml) of the probands and their parents by using Tiangen DNA extraction kit (Tiangen Biotech, China). DNA quality was quantified with a NanoDrop 2000 (Thermo, USA).

### Whole Exome Sequencing and Bioinformatics Analysis

WES was carried out using an Agilent SureSelect Human All Exon V6 Kit (Agilent Technologies Inc., USA) on an Illumina NovaSeq 6000 platform (Illumina Inc., CA, USA). Data and bioinformatic analyses were performed according to a method described by a previous study (Luo et al., 2019). Candidate variants were confirmed in the parents in each family by Sanger sequencing. PCR products were bi-directionally sequenced using the BigDye Terminator v3.1 Cycle Sequencing Kit (Applied Biosystems, USA) on an ABI 3500DX Genetic Analyzer (Applied Biosystems) after purification on 2% agarose gels.

Variants were described according to the nomenclature recommended by the Human Genome Variation Society (www.hgvs.org/). Variants were annotated using ANNOVAR (https://annovar.openbioinformatics.org/en/) and filtered according to their predicted effects and allele frequencies in the public database gnomAD (http://gnomad.broadinstitute.org/). Novel variants were checked in the Human Gene Variant Database (www.hgmd.cf.ac.uk) and ClinVar database (www.ncbi.nlm.nih.gov/clinvar/). InterVar (http://wintervar.wglab.org/) software was used to evaluate the pathogenicity of all variants according to the standards and guidelines of the American College of Medical Genetics and Genomics (Li and Wang, 2017).

### Methylation Detection

The detection of methylation of *KCNQ1OT1* gene was performed by using the SALSA MLPA Probemix ME030 BWS/RSS kit (LOT: B2-1110, MRC Holland, Netherlands). The experimental operation was performed according to the protocol provided by the manufacturer. Methylation of *KCNQ1OT1* gene was analyzed by the Coffalyser software (MRC-Holland, Netherlands).

## Results

### Phenotype Spectrum Description

Among the 260 probands analyzed, a genetic diagnosis was found in 135 patients (Supplementary Table S1). A total of 188 phenotypes were detected in these 135 positive/likely positive probands (Supplementary Table S2). Epilepsy was diagnosed in all 135 patients, and EEG abnormalities were detected in 21 patients. Additionally, the following symptoms were reported: tetany (18 patients), intellectual disability (13 patients), motor deterioration (11 patients), and delayed speech and language development (nine patients) (Supplementary Table S2). Moreover, status epilepticus and epileptic encephalopathy exhibited high frequencies, as both were found in five patients (Supplementary Table S2). In the undiagnosed patients, total 121 phenotypes were detected (Supplementary Table S2). Similar to the diagnosed patients, epilepsy was detected in all 125 undiagnosed patients. Although there are some differences in the order of phenotype distribution, the main phenotypes are tetany (23 patients), EEG abnormality (10 patients), delayed speech and language development (10 patients) (Supplementary Table S2).

### Variant Analysis

Three modes of inheritance were observed among the 135 positive/likely positive cases, including 105 autosomal dominant (AD) cases, 16 autosomal recessive (AR) cases, and 15 X-linked cases (Supplementary Table S1). Eighty-one epilepsy-related genes were detected; the gene most frequently detected was *KCNQ2* (nine times), followed by *PRRT2* (seven times), *SCN1A* (six times), *SCN2A* (five times), *SPTAN1* (four times), and *TSC2* (four times).

Four patients suffered from two diseases, and variants in two genes were detected. Patient 20 suffered from both Charge syndrome and susceptibility to juvenile myoclonic epilepsy-1, the patient 25 suffered from both autosomal dominant intellectual developmental disorder 50 and benign familial neonatal infantile seizures 3/early infantile epileptic encephalopathy 11, patient 43 suffered from both familial focal epilepsy with variable foci-1 and Pendred syndrome and patient 112 suffered from both Beckwith-Wiedemann syndrome and mucopolysaccharidosis II (MPS II) (Supplementary Table S1).

**FIGURE 1:**
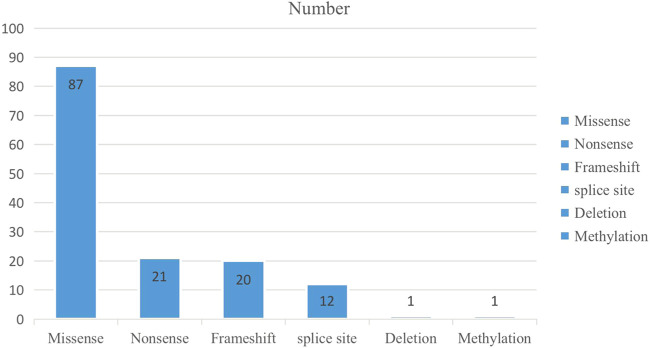
The number of the variant type in 135 positive/likely positive probands.

In total, 142 variants of 81 genes were detected among the 135 positive/likely positive probands. These variants included five variant types, including 87 missense variants (61%), 21 nonsense variants (15%), 20 frameshift variants (14%), 12 splice site variants (8%), one methylation variant (1%), and one deletion variant (1%) (Figure 1; Supplementary Table S1).

Among these 142 variants, 87 of 66 genes were novel, accounting for 61.3% (87/142) of the variants (Supplementary Table S3). According to the American College of Medical Genetics guidelines and InterVar software, 31 novel variants were categorized as “pathogenic”, 51 were “likely pathogenic” and five splicing variants were “Uncertain significance” (Supplementary Table S3). We use Alamut Visual Plus software (Interactive Biosoftware, Switzerland) to analyze the pathogenicity of these five splice site variants: *GNAO1(NM_138736.3)* c.877+5A>G, *KCNQ2(NM_172107.4)* c.1632-5T>A, *KIF4A(NM_012310.5)* c.2232+3A>G, *SCN3A(NM_006922.4)* c.1173+5G>A, *SPTAN1(NM_001363759.2)* c.3520-7C>T. And all these five splicing variants may affect the splicing function (Supplementary Table S4).

### Phenotype-Gene Correlation

Among the 135 positive/likely positive probands, 106 patients had more than two phenotypes, and 67 patients had more than three phenotypes. Among patients with only one disease-causing gene, *MMUT*, *KMT2A*, *MECP2*, *HIVEP2*, *TSCE*, *KCNQ2*, *POLG2*, *SYNGAP1*, *DGUOK*, *GALC*, *ARX*, *ADNP*, *COL3A1*, and *SCN2A* were responsible for the most phenotypes in patients.

In these 135 positive/likely positive probands, which was caused by *ADGRV1*, *CACNA1H*, *CHD8*, *GNAO1*, *HECW2*, *KCNQ2*, *KMT2A*, *KMT2C*, *MECP2*, *NAA15*, *SCN1A*, *SCN2A*, *SETBP1*, *SLC2A1*, *SMC1A*, *STXBP1*, *SYNGAP1* and *TSC2* et al. are accompanied by the intellectual disability (Supplementary Table S1). Syndromes with epilepsy are caused by *ADNP*, *ARID1A*, *CASR*, *CHD7*, *COL3A1*, *DGUOK*, *KCNK4*, *KMT2A*, *KMT2C*, *MECP2*, *NIPBL*, *PIGO*, *SETBP1* and *UBE3A* et al. (Supplementary Table S1). Epilepsy caused by the *KCNQ2*, *KCNQ3*, *PRRT2*, *SCN2A* and *SCN8A* gene are relatively benign with mild symptoms and consequences.

We performed pathway analysis of 81 pathogenic genes detected in the 135 positive/likely positive patients. Forty-four genes were enriched in the Kyoto Encyclopedia of Genes and Genomes database by the Database for Annotation, Visualization and Integrated Discovery tool (https://david.ncifcrf.gov/). These genes were located in 14 pathways, with the cholinergic synapse, dopaminergic synapse, and MAPK signaling pathways containing the most genes, followed by circadian entrainment and the glutamatergic synapse pathway (Supplementary Table S5). Some genes were involved in more than one pathway, including *GNAO1*, *GRIN2A*, *CACNA1A*, and *GNB5*. Among them, *GNAO1* was involved in the most pathways (Supplementary Table S5).

## Disscusion

In this study, we performed genetic analysis of 260 infants/children with epilepsy. The positive detection rate of our study was 51.9% (135/260), including four patients who suffered from two diseases. In total, 142 variants of 81 genes were detected in 135 patients (Supplementary Table S1). 87 of the 142 epilepsy-causing variants located in 66 genes had not been previously reported (Supplementary Table S3).

Zhang et al. (2015) performed variant analysis using panel-targeted next-generation sequencing in a cohort of 253 Chinese children with unexplained epilepsy. Their detection rate was 26% (Zhang et al., 2015), which was similar to that found by Kothur et al. (2018). In general, the diagnostic yields of targeted panels of 35–265 genes ranged between 10% and 48.5% (Kothur et al., 2018). The reason for the low detection rate may be that they used panel sequencing, which includes fewer genes, instead of WES. When using WES, the diagnostic yield ranged from 11% to 72% (Epi, 2013; Veeramah et al., 2013; Michaud et al., 2014; Helbig et al., 2016). In our study, the detection rate was 52.7%, which suggests that WES may be an effective tool for identifying the causative molecular factors in patients (Helbig et al., 2016). In our study, four patients (patient 20, 25, 43 and 112) suffered from two diseases, considering that epilepsy patients may suffer from two or more diseases, WES or whole-genome sequencing is a good diagnostic strategy.

Among our 135 positive/likely positive patients, variants were identified in a total of 81 unique genes, 27 of which were found in two or more unrelated patients (Table 2). Variants in 12 genes (*KCNQ2, PRRT2, SCN1A, SCN2A, SPTAN1, TSC2, ADGRV1, KMT2C, PCDH19, RARS2, SCN8A,* and *SLC2A1*) were found most frequently, with variants in each of these genes identified in three or more unrelated probands (Table 2). Variants were most frequently detected in *KCNQ2*, which has not been systematically investigated in existing studies; however, as a supplement to the findings of Helbig et al. (2016), our study may help clarify the genetic characteristics of patients with this patient population.

**TABLE2:** The epilepsy related genes and frequency detected in this study.

Gene	Frequency	Gene	Frequency	Gene	Frequency
*ACADS*	1 (0.73%)	*GLRA1*	1 (0.73%)	*NIPBL*	1 (0.73%)
*ACADVL*	1 (0.73%)	*GNAO1*	1 (0.73%)	*NPRL3*	1 (0.73%)
*ADGRV1*	3 (2.19%)	*GNB5*	1 (0.73%)	*PCDH19*	3 (2.19%)
*ADNP*	1 (0.73%)	*GRIN2A*	1 (0.73%)	*PIGO*	1 (0.73%)
*AIMP1*	1 (0.73%)	*GRIN2B*	1 (0.73%)	*POLG2*	2 (1.46%)
*ALDH7A1*	1 (0.73%)	*HECW2*	1 (0.73%)	*PRRT2*	7 (5.11%)
*ALG13*	1 (0.73%)	*HIVEP2*	1 (0.73%)	*PURA*	1 (0.73%)
*ARID1A*	1 (0.73%)	*HK1*	1 (0.73%)	*RARS2*	3 (2.19%)
*ARX*	2 (1.46%)	*HNRNPU*	2 (1.46%)	*SCN1A*	6 (4.38%)
*BCKDHB*	1 (0.73%)	*IDS*	2 (1.46%)	*SCN1B*	1 (0.73%)
*CACNA1A*	2 (1.46%)	*IQSEC2*	1 (0.73%)	*SCN2A*	5 (3.65%)
*CACNA1E*	1 (0.73%)	*KCNA2*	1 (0.73%)	*SCN3A*	2 (1.46%)
*CACNA1H*	2 (1.46%)	*KCNB1*	1 (0.73%)	*SCN8A*	3 (2.19%)
*CACNB4*	1 (0.73%)	*KCNK4*	1 (0.73%)	*SCN9A*	2 (1.46%)
*CASR*	1 (0.73%)	*KCNQ2*	9 (6.57%)	*SETBP1*	2 (1.46%)
*CHD2*	2 (1.46%)	*KCNQ3*	1 (0.73%)	*SLC2A1*	3 (2.19%)
*CHD8*	1 (0.73%)	*KCNT1*	2 (1.46%)	*SLC6A1*	1 (0.73%)
*CHRNB2*	1 (0.73%)	*KIF4A*	1 (0.73%)	*SLC6A5*	1 (0.73%)
*CNKSR2*	1 (0.73%)	*KMT2A*	1 (0.73%)	*SMC1A*	1 (0.73%)
*COL3A1*	1 (0.73%)	*KMT2C*	3 (2.19%)	*SPTAN1*	4 (2.92%)
*CUX2*	1 (0.73%)	*KRIT1*	1 (0.73%)	*STX1B*	1 (0.73%)
*DEPDC5*	2 (1.46%)	*MBD5*	1 (0.73%)	*STXBP1*	1 (0.73%)
*DGRV1*	1 (0.73%)	*MECP2*	2 (1.46%)	*SYNGAP1*	1 (0.73%)
*DGUOK*	1 (0.73%)	*MED17*	1 (0.73%)	*TSC1*	1 (0.73%)
*EFHC1*	2 (1.46%)	*MMUT*	1 (0.73%)	*TSC2*	4 (2.92%)
*FLNA*	1 (0.73%)	*NAA15*	1 (0.73%)	*UBA5*	1 (0.73%)
*GALC*	1 (0.73%)	*NF1*	2 (1.46%)	*UBE3A*	1 (0.73%)

Among our 135 positive/likely positive probands, 143 variants of 81 genes were detected, including five variant types (Figure 1; Supplementary Table S1) including 87 novel variants (Supplementary Table S3). Among these novel variants, 50 were missense variants (57.5%), 11 were nonsense variants (12.6%), 17 were frameshift variants (19.5%), eight were splice site variants (9.2%), and one was a deletion variant (1.1%). Similarly, novel *KCNQ2* variants exhibited the highest frequency, followed by *RARS2* and *SPTAN1* variants. *RARS2* causes pontocerebellar hypoplasia type 6 (PCH6), which is characterized by an abnormally small cerebellum and brainstem and associated with severe developmental delays (Edvardson et al., 2007). Most PCH6 patients have seizures (Edvardson et al., 2007; Rankin et al., 2010; Li et al., 2015); however, some only have cerebellar hypoplasia (Edvardson et al., 2007). Among our three patients with PCH6 caused by *RARS2* variants, all simultaneously showed both cerebellar hypoplasia and seizures. *SPTAN* is the causative gene of developmental and epileptic encephalopathy-5, which is a neurologic disorder characterized by global developmental delays and the onset of tonic seizures. The seizures often tend to be refractory to treatment, and EEG shows hypsarrhythmia, consistent with a clinical diagnosis of West syndrome. Affected individuals have severely impaired psychomotor development with lack of visual attention, poor head control, feeding difficulties, microcephaly, and spastic quadriplegia. Our three developmental and epileptic encephalopathy-5 patients all exhibited refractory epilepsy and lack of visual attention.

The incidence of inherited metabolic diseases (IMDs) was low; however, the estimated collective prevalence is 1:1,000, and more than 200 IMD patients have been reported to present with seizures (Wolf et al., 2005; Mercimek-Mahmutoglu et al., 2015). In our study, five patients were diagnosed with IMDs and had seizures as a symptom (Table 2). Because the majority of IMDs have disease-specific treatments, it is very important to carry out IMD screening in patients with epilepsy.

We performed pathway analysis of 81 pathogenic genes detected in the 135 positive/likely positive patients. Forty-four genes were enriched in the Kyoto Encyclopedia of Genes and Genomes database. These genes were located in 14 pathways, with the cholinergic synapse, dopaminergic synapse, and MAPK signaling pathways containing the most genes, followed by circadian entrainment and the glutamatergic synapse pathway (Supplementary Table S5). Some of these genes play a role in more than one pathway; among them, *GNAO1* plays a role in the most pathways (Supplementary Table S5). Moreover, this analysis method may be beneficial for identifying possible pathogenic genes in epilepsy negative cases.

In our study, AD variants explained 77.8% (105/135) of positive/likely positive findings in the patient cohort; AR variants were responsible for approximately 11.9% (16/135), and X-linked variants explained 10.3% (15/135) of positive/likely positive cases (Supplementary Table S1). Among X-linked cases, 10 were X-linked recessive (XLR) and six were X-linked dominant (XLD). Developmental and epileptic encephalopathy-9 caused by variants in PCDH19 is an XLR disease. This disorder affects only heterozygous females, and transmitting males are unaffected (Jamal et al., 2010), but genetic counseling is needed for these individuals. Taken together, our findings suggest that AD/XLD inheritance may explain approximately 82.2% of our cases of infant and childhood epilepsy, and AR/XLR inheritance may explain approximately 17.8% of our cases.

Epilepsy was found in all of our 135 positive/likely positive probands, and EEG abnormalities (21 patients), tetany (18 patients), intellectual disability (13 patients), motor deterioration (11 patients), and delayed speech and language development (nine patients) are the more frequent phenotypes (Supplementary Table S2). In the undiagnosed patients, similar to the diagnosed patients, tetany (23 patients), EEG abnormality (10 patients), delayed speech and language development (10 patients) are also the more frequent phenotypes (Supplementary Table S2). These results show that in the treatment of patients with epilepsy, attention should be directed to avoiding intellectual disability, motor retardation, and language retardation.

Among the 135 positive/likely positive probands, 67 patients had more than three phenotypes, indicating that epilepsy has a highly heterogeneous clinical phenotype. *MMUT*, *KMT2A*, *MECP2*, *HIVEP2*, *TSCE*, *KCNQ2*, *POLG2*, *SYNGAP1*, *DGUOK*, *GALC*, *ARX*, *ADNP*, *COL3A1*, and *SCN2A* were responsible for the most phenotypes in patients. More comprehensive treatment may be needed to reduce the impact of diseases caused by these genes on patients. Although epilepsy caused by the *KCNQ2*, *KCNQ3*, *PRRT2*, *SCN2A* and *SCN8A* gene are relatively benign, in our study, more than 18 genes caused epilepsy accompanied by the intellectual disability and more than 14 genes caused syndromes with epilepsy (Supplementary Table S1). Therefore, genetic testing is a very important prerequisite for precise intervention in epilepsy patients.

Our study also has some limitations. Previous studies showed that copy number variation (CNV) is also a cause of epilepsy (Scheffer et al., 2010; Mefford et al., 2010). However, in our study, because of technical limitations, we did not perform CNV analysis on patients, which might prevent diagnosis of some patients with CNVs. We found five novel splicing variants, we only performed function prediction, but did not perform minigene function verification.

## Conclusion

In summary, our findings extend the variant spectrum of genes related to epilepsy. Since some epilepsy is accompanied by developmental delay, intellectual disability and some serious syndromes, timely molecular genetic testing is very important for the treatment of patients, and WES or whole-genome sequencing is a good diagnostic strategy for identifying the causative molecular factors in epilepsy patients. All newborns should be recommended to be screened for genetic metabolic diseases to avoid epileptic seizures, mental retardation and other diseases caused by genetic metabolic diseases. Our results will be helpful for genetic testing, clinical medication and genetic counseling in epilepsy for patients with epilepsy.

## Data Availability

The datasets for this article are not publicly available due to concerns regarding participant/patient anonymity. Requests to access the datasets should be directed to the corresponding authors.
